# General description and understanding of the nonlinear dynamics of mode-locked fiber lasers

**DOI:** 10.1038/s41598-017-01334-x

**Published:** 2017-05-02

**Authors:** Huai Wei, Bin Li, Wei Shi, Xiushan Zhu, Robert A. Norwood, Nasser Peyghambarian, Shuisheng Jian

**Affiliations:** 1 Key Laboratory of All-Optical Networks and Advanced Communication Networks of Ministry of Education, Beijing Jiaotong University, Beijing, 100044 China; 2 Institute of Light Wave Technology, Beijing Jiaotong University, Beijing, 100044 China; 3 Department of Communication Engineering, Communication University of China, Beijing, 100024 China; 4 College of Optical Sciences, University of Arizona, Tucson, Arizona 85721 USA; 5 College of Precision Instrument and Optoelectronics Engineering, Tianjin University, Tianjin, 300070 China; 6 NP Photonics, Tucson, AZ 85747 USA

## Abstract

As a type of nonlinear system with complexity, mode-locked fiber lasers are known for their complex behaviour. It is a challenging task to understand the fundamental physics behind such complex behaviour, and a unified description for the nonlinear behaviour and the systematic and quantitative analysis of the underlying mechanisms of these lasers have not been developed. Here, we present a complexity science-based theoretical framework for understanding the behaviour of mode-locked fiber lasers by going beyond reductionism. This hierarchically structured framework provides a model with variable dimensionality, resulting in a simple view that can be used to systematically describe complex states. Moreover, research into the attractors’ basins reveals the origin of stochasticity, hysteresis and multistability in these systems and presents a new method for quantitative analysis of these nonlinear phenomena. These findings pave the way for dynamics analysis and system designs of mode-locked fiber lasers. We expect that this paradigm will also enable potential applications in diverse research fields related to complex nonlinear phenomena.

## Introduction

As ideal ultra-short pulse sources, mode-locked lasers, particularly fiber based mode-locked lasers, have generated great interest because of their inherent advantages and attractive properties^1–3^. The interplay among the many factors (nonlinear, dispersion, and positive and negative feedback) in the cavity results in the rich and complex nonlinear dynamics of the pulses in mode-locked fiber lasers. Typical pulse shapes include sech^2^ ^2^, parabolic (self-similar)^4–6^ and flat top dissipative soliton resonance (DSR)^7–12^. The working states of the laser can include single-pulse state^2^, multi- pulse state^13–16^, Q-switched mode-locking state^1^, and unstable pulses with periodic or non-periodic fluctuation state^17–27^. Moreover, stochastic phenomena, hysteresis and multistability^28–31^ further exemplify the complexity of the dynamics of mode-locked fiber lasers. The broad range of temporal scales (from the femtosecond scale for pulse detail to the millisecond scale for Q-switched envelope fluctuations) presents significant difficulties in the analysis of mode-locked lasers. The description, understanding and control of the complex dynamics governing the behaviour of mode-locked fiber lasers require further study. Extensive effort has been devoted to the understanding of mode-locked fiber lasers, fundamental equations have been developed^2, 32–34^, new types of pulses have been discovered^4–12^, and multi-pulse phenomena have been analysed^13–16^, However, the relevant nonlinear partial differential equations can usually be solved only by numerical methods (particularly for multi-pulse states and unstable pulse states)^33–35^. If the time resolution is high and time span is excessively large, the numerical simulation will require a relatively large amount of computing resources. Moreover, because of the complex nonlinear interactions, stochastic character and initial value sensitivity^28–31^, it is difficult to clearly and efficiently analyse the roles of various factors. There remains a lack of an ideal framework with convenient mathematical representations and clear physical meaning that can be used to provide a unified description for the various states of a mode-locked laser and to determine the driving force behind the complex nonlinear behaviour observed. In particular, the lack of a mathematical analysis tool for quantitative analysis is a fundamental limitation.

From a methodological point of view, most current models attempt to study complex nonlinear phenomena using the traditional reductionism approach. Undoubtedly, many important and useful results have been derived from models based on this approach. However, explaining all complex nonlinear phenomena with this approach is inefficient and impractical. It is difficult to develop clear physical insight, particularly for macroscopic phenomena emerging from highly structured complex nonlinear behaviour^36–39^.

Complexity theory is becoming a powerful approach to address a wide range of problems in chemistry, biology, economics and geomorphology^36–39^. Here, we use this methodology to analyse the dynamics of mode-locked fibre lasers.

In this paper, by implementing hierarchy and the multi-scale method, the problem can be simplified from an infinite dimensional problem into an iterative mapping with finite but changeable dimensionality. We find that the complex dynamical behaviours of mode-locked lasers are actually manifestations of various attractors with different dimensions under various conditions. In addition, this theoretical framework can help us reveal the origin of complex dynamical behaviour and provide quantitative analysis of many nonlinear phenomena, such as stochastic phenomena, hysteresis and multistability. We find that multi-attractors, fluctuations, and the variation of attractor basins according to control parameters are the core factors for these phenomena, the mechanism of which will be discussed in detail in this article.

## Hierarchical structure, the coarse-grain method and the phase space

**Figure 1:**
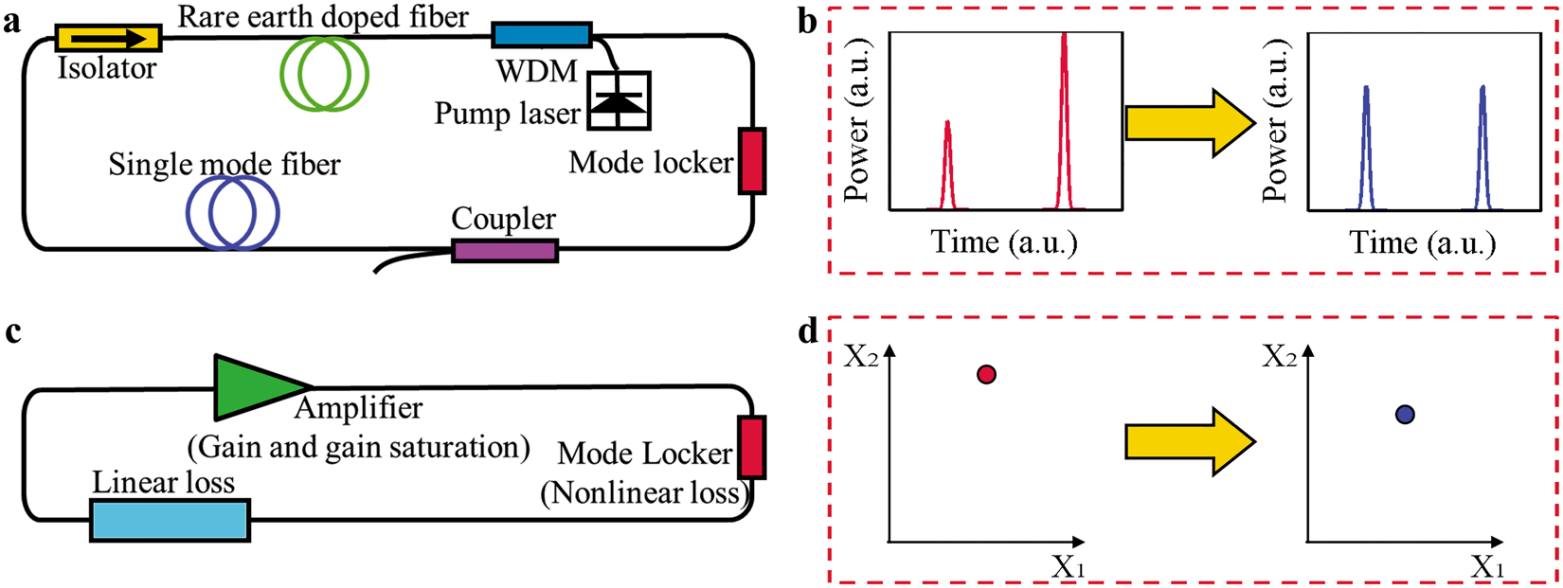
Schematic diagram of the mode-locked laser.

**Figure 2:**
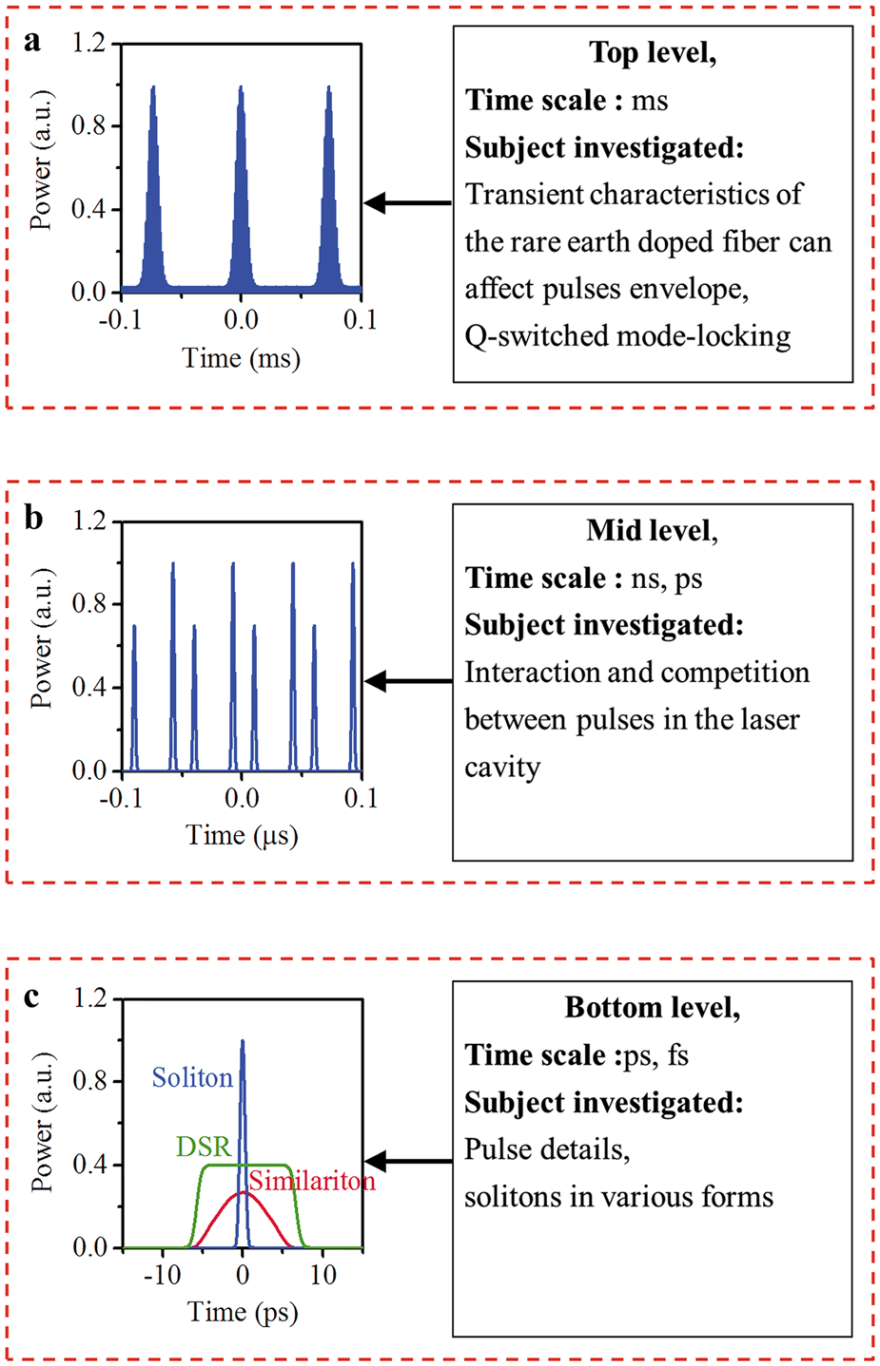
The hierarchy model used to analyse the dynamics of the mode-locked laser. (Variables characterizing the system dynamics are arranged by temporal scale).

The mode-locked laser is an infinite dimensional dynamical system with a feedback structure (see Fig. 1(a)) and constitutes a complex nonlinear system^40, 41^. Large composite systems, despite their complexity on the small scale, sometimes crystallize into large-scale patterns that can be conceptualized relatively simply^36^. When focused on these macroscopic phenomena, we can divide the complex system into several levels. Using the hierarchical and coarse-grain method, we can observe that nonlinear dynamical phenomena emerge at the macro level, which allows key aspects of the system to be separated from extraneous details^36–41^. The mode-locked laser is such a system, with a hierarchical structure that can be naturally divided into three levels. (see Fig. 2).

Because there have been many reports in the literature on models of the pulse details (for a review article, see, e.g., ref. 7) and because of space limitations, we will not analyse this level. As a bridge to connect micro- and macro-phenomena, the intermediate-level is a crucial part of the hierarchical model and will be our primary focus. The top-level view and interactions among the various levels will be discussed concisely near the end of this paper. The pulses in a mode-locked laser exhibit quantization^13, 14^, which allows a simplified description of the mid-temporal scale level. We ignore the details of the pulse, i.e., a pulse is considered a basic unit (a grain). The existing model^16^ gives a geometrical description of the onset of multi-pulsing in mode-locked laser cavities, and as such are embryonic coarse-grain models. We have improved upon this description and incorporate it within a hierarchical model. Next, as an important step, we employ “dimensions” and “phase space” concepts to provide a deeper analysis, as will be explained in detail below. The pulse energy *E*_*j*_ (the subscript “*j*” is the number index of the pulse in the laser cavity) is determined by the equivalent width *t*_*j*_ (pulse duration) and equivalent amplitude *x*_*j*_ (Eq. (1)). In the simplest case, the pulse energy is independent of the pulse duration, and the picture reduces to the model based on pulse energy, as in ref. 16. In the more complex case, if the pulse amplitude is relevant to the pulse duration, then Eq. (2) should be used. Gain saturation has an influence on the average laser power, as determined by the pulse number in the laser cavity and the individual pulse energies (Eqs (3 and 4)). In addition, nonlinear loss attributable to the mode locker affects each pulse. There are many types of models can be used to describe the effect of the mode locker (*f*(*x*_*j*_) in Eq. (5))^16, 42–44^.

1 $${E}_{j}=\int {p}_{j}dt={x}_{j}{t}_{j}={x}_{j}{t}_{eff}$$

2 $${t}_{eff}=T(p)$$

3 $${\rm{G}}={e}^{g}$$

4 $${\rm{g}}=\frac{{g}_{0}}{1+\frac{{\sum }_{j=1}^{m}{E}_{j}}{{E}_{sat}}}$$

5 $${{\rm{L}}}_{j}=f({x}_{j})$$

Two types of mode lockers are widely used in mode-locked fiber lasers: one type is based on saturable absorption. (e.g., the semiconductor saturable absorber mirror (SESAM), and absorbers constructed from carbon-based materials, such as single wall carbon nanotubes (SWCNTs) and graphene). The other type of mode locker is based on nonlinear polarization rotation (NPR). Although the fiber lasers with a mode locker based on saturable absorption (e.g., CNT-based absorbers) are commonly independent of the polarization of laser and have high environmental stability^44^, here, we focus on the NPR mode locker because in addition to its high power tolerance, the nonlinear property (nonlinear loss) of the NPR mode locker is more complex than that of absorbers based on saturable absorption. When the input power is increased, the sign of the feedback (e.g., whether it is positive or negative) can be changed. This variation causes such lasers to exhibit more abundant nonlinear phenomena. Another advantage of this mode-locking method is that, unlike the absorbers based on saturable absorption, for which the properties cannot be changed after production, we can change the nonlinear loss property by adjusting the components of the NPR mode-locking lasers (e.g., the polarizer and polarization controller). In addition, we can use multiple transmission filters to engineer the nonlinear loss property^42, 45^.

The coarse-grain method transforms an infinite dimensional dynamical system (Fig. 1(a,b)) into a discrete dynamical system generated by an iterated map of countable dimensions (Fig. 1(c,d)). The phase space for the mode-locked laser system is an R^m^ space, where a vector in R^m^ space can be used to represent a state at a given moment in time in a mode-locked laser system. Every component of the m-dimensional vector is a representation of the equivalent power of each pulse. The dimension “m” corresponds to the number of pulses in the laser cavity.

The intra-cavity pulse number, which is our primary focus, reflects the competition and influence among the pulses. This behaviour can be described and analysed by a high-dimensional discrete dynamical system. The time-dependent states of the pulses can be expressed as a trajectory in the phase space. Note that the “m” in the m-dimensional R^m^ space is neither infinite nor invariant, as will become clear below. Changes in the laser parameters are often accompanied by extension or collapse of the dimensions in phase space; mathematically, this transformation constitutes a mapping with changeable dimensionality.

## Attractors: the existence states of pulses in mode-locked lasers

Relative to common nonlinear systems, mode-locked lasers exhibit more interesting and unique characteristics based on their dimensionality.

**Figure 3:**
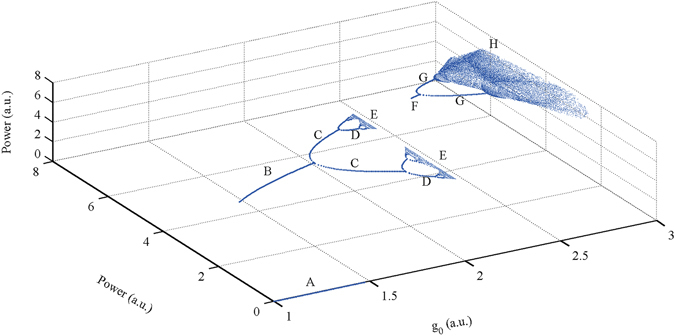
Bifurcation orbit diagram in multi-dimensional phase space under certain initial conditions with different gain coefficient g_0_. Point A (1 < g_0_ < 1.46), there is no pulse; point B (1.46 < g_0_ < 2.02), stable single pulse state; point C (2.02 < g_0_ < 2.32) and D (2.32 < g_0_ < 2.40), single pulse with periodic fluctuations; point E (2.43 < g_0_ < 2.47), single pulse that exhibits chaotic fluctuation; point F (2.47 < g_0_ < 2.53), stable double pulse state; point G (2.53 < g_0_ < 2.74), double pulse with periodic fluctuations; point H (g_0_ > 2.82), chaotic fluctuation.

The attractor shape and topological structure are not the only origins of attractor diversity, as dimensionality also plays an important role. Consider Fig. 3 as an example. When g_0_ is too low (g_0_ < 1.46), there is no pulse (Fig. 3, point A). Under the appropriate conditions for the gain coefficient (1.46 < g_0_ < 2.02), the laser operates stably in the single-pulse state, and the attractor is a fixed point in phase space (Fig. 3, point B). At points C (2.02 < g_0_ < 2.32) and D (2.32 < g_0_ < 2.40) in Fig. 3, the attractor becomes two (or more) discrete points, i.e., the operating state is a single pulse with periodic fluctuations. At point E (2.43 < g_0_ < 2.47) in Fig. 3, the attractor is a set of discrete points, i.e., a strange attractor in 1 dimension. In other words, there is a single pulse that exhibits chaotic fluctuation. At point F (2.47 < g_0_ < 2.53), the attractor is once again a fixed point. However, different from point B, point F is a point in 2-dimensional space. At points G (2.53 < g_0_ < 2.74) and H (g_0_ > 2.82) in Fig. 3, periodic orbits and chaos attractors emerge in 2D phase space.

In fact, when increasing the gain coefficient to higher values, we can identify attractors in higher-dimensional space (Supplementary Videos S1–S3). In these cases, however, the bifurcation diagram becomes a four- (or even higher) dimensional graph from which it is difficult to provide an intuitive graphical representation.

Even these simple examples can provide many physical insights regarding the dynamics of mode-locked lasers. An attractor is generally a set of points in the phase space; thus, for a given parameter g_0_ and a phase space with a sufficiently large dimensionality (as an initial condition), all of the points of the attractor will finally be located in a sub-space with limited dimensionality (Supplementary Video S3). For different values of the parameter g_0_, the dimensionality of the sub-space may be different. In other words, the variation of the gain coefficient can cause extension or collapse of the dimensions in phase space (Supplementary Videos S1 and S3), showing that increasing dimensionality can provide space for increasing complexity. In this process, fluctuations seed the creation of new dimensions, and the control parameter (gain coefficient) determines the dimensionality of the attractor.

By using the coarse-grain method, we observe that the behaviour of complex systems can be successfully modelled using a small number of macroscopic quantities. Such macroscopic observables play the role of a thermodynamic order parameter in the synergetic framework^46–49^. Here, the phase point in the multi-dimensional mapping phase space with variable dimension represents the state of the mode-locked laser. Switching of the laser operating states by changing the control parameter can be interpreted as the order parameter switching between attractors in the phase space. Dimension mutation of this parameter (extension or collapse of the dimensions) occurs at the bifurcation points on the bifurcation diagram (Supplementary Videos S1 and S3), essentially acting in a manner similar to a phase transition process (i.e., a nonequilibrium phase transition). We find that variation of the attractor basin is the driving force for this transition process and that the random fluctuation acts to seed the transition.

**Figure 4:**
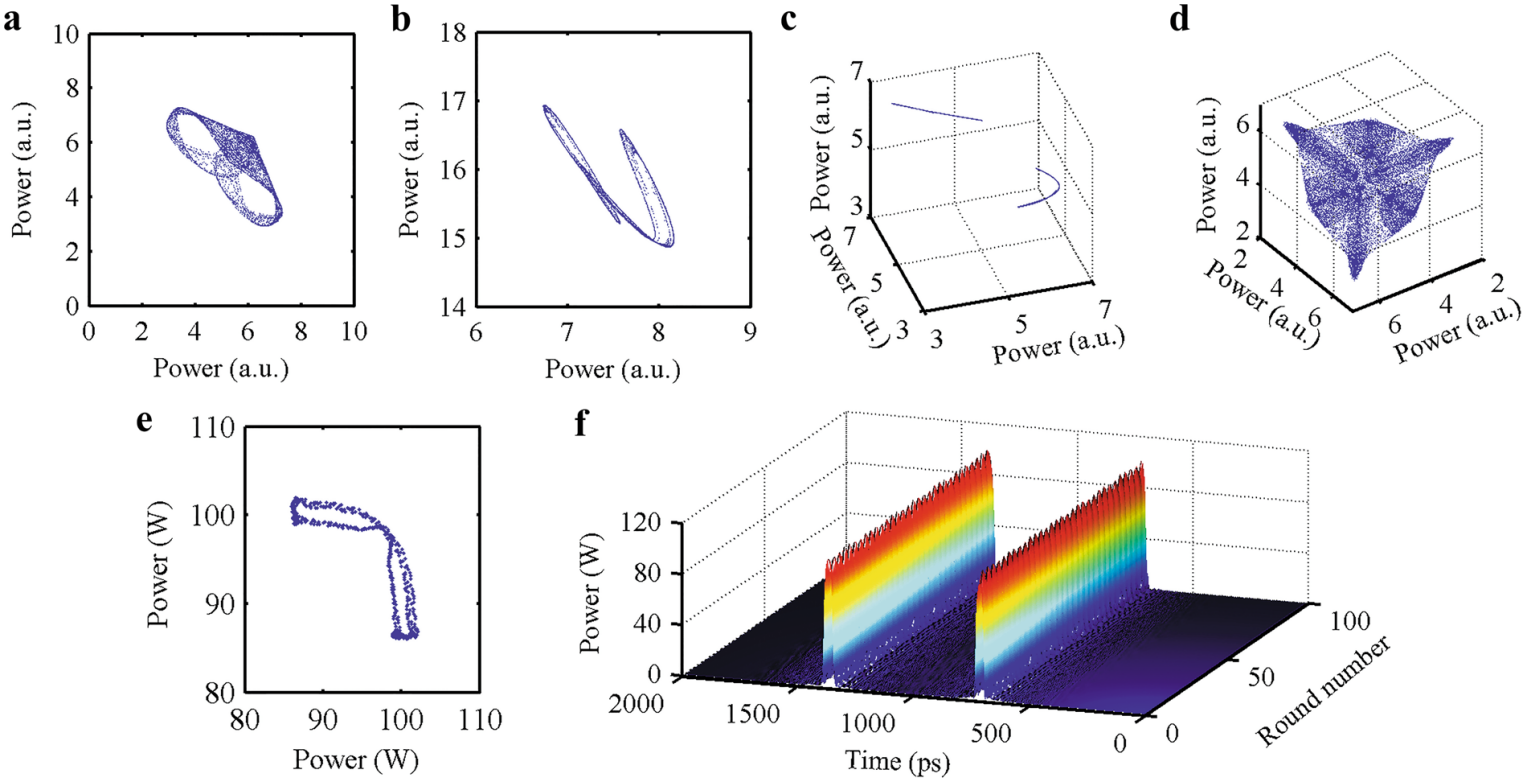
Chaos attractors in phase space. (**a**,**b**) shows the attractor for two pulses. (**c**,**d**) shows the attractor for three pulses. (**e**,**f**) shows the result derived by the full numerical model.

Complex and diverse operating states can be presented by attractors with various styles and shapes. The strange attractors exist in a variety of forms, as shown in Fig. 4 and Supplementary Videos S4–S7. Supplementary Table S1 summarizes and lists the operating states of a mode-locked laser. Note that using the model and methodology we have provided, we can identify a state for pulses in mode-locked lasers, namely, coexisting multi-pulses that form a chaos attractor in phase space. Figure 4 shows the attractors for two pulses and three pulses (Supplementary Videos S4–S7). The Lyapunov exponent (Supplementary Fig. S1) and correlation dimension^50–52^ (Supplementary Fig. S2 and S3) confirm the existence of strange attractors.

The existence of the chaos attractor is also confirmed using a traditional direct numerical model. Figure 4(e,f) and Supplementary Video S10 show the pulse in the time domain and the Poincaré sections of the pulses (taken at the peak power of pulses) in phase space derived by direct numerical simulation.

According to experimental observations^29, 31^, an unstable mode-locking state is found when adjusting the gain parameter to change the number of pulses inside the cavity. “Chaotic behaviour between single- and multi-pulse operation”^29^ and “alternately evolving on the stable and unstable states”^31^ are reported. The reported experimental phenomena can be explained by the attractor changing from “a fixed point” to “a strange attractor” and then to a new “fixed point” in higher dimension space in our theory, as discussed above (see Fig. 3).

In addition to the previously mentioned unstable states, there have also been some experimental observations of chaos phenomena in mode-locked fiber lasers^20, 53, 54^. This paper provides a potential theoretical explanation for the emergence of chaos phenomena and other unstable states^23–27^.

**Figure 5:**
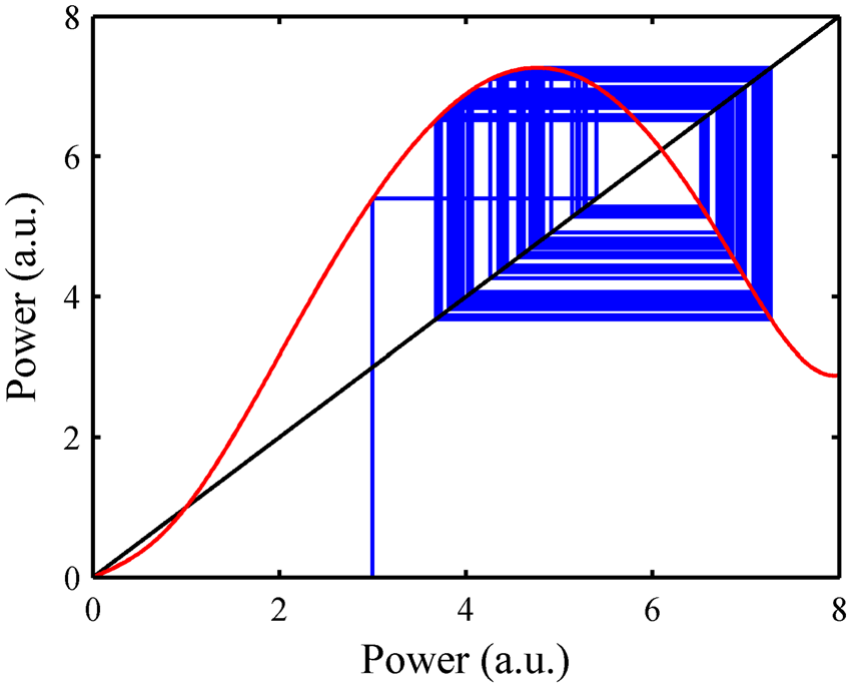
Orbit diagram for a chaos pulse in the laser. Red line: Nonlinear mapping function of the laser; Blue line: Orbit diagram of the pulse; Black line: x = y.

The mechanism of the chaotic motions of multiple intra-cavity pulses is as follows. The optical pulse passes through the mode locker and the amplifier in the laser cavity. The nonlinear loss and gain characteristics of the amplifier form a feedback loop together; mathematically, they form a nonlinear mapping. The loop of the laser in the cavity corresponds to the iteration of the mapping. When the nonlinear mapping function has a large negative slope, the laser will be more likely to enter the chaotic state (Fig. 5 shows the orbit diagram for a chaotic pulse in the laser, which is similar to the case of logistic mapping^16, 40^. This effect causes the power of the pulses in the cavity to fluctuate. It is an important factor leading to chaos. For the case of multiple pulses, another factor is that pulses can interact with each other through amplifier gain (Eq. (4) and the transient model discussed in a later section “Outlook and discussions”). According to the sensitive dependence on initial conditions (SDIC), a.k.a., the so-called “butterfly effect”, the fluctuation of a pulse can have an impact on the trajectory of other pulses. Thus, the multi-pulse chaotic situation becomes more complex and evolves to the high-dimensional chaotic attractor, as previously discussed.

## Attraction basin, the mechanism behind stochastic phenomena, hysteresis and multistability

In the previous section, we analysed the operating states of the mode-locked laser. In this section, we will discuss another important problem: the dynamics of conversion between these states, including state transition and pulse start-up. There are many complex phenomena (stochastic phenomena, hysteresis, and multistability, among other phenomena) involved in these dynamics^28–31^. However, the mechanisms behind many of these phenomena remain obscure.

For a thorough grasp of the state transition problem, we need a global understanding of the laser’s behaviour under all possible initial conditions. Direct numerical modelling^55^ would require an enormous amount of computation and would suffer from many uncontrollable factors (e.g. pulse splitting, pulse deformation and amplified spontaneous emission (ASE) noise). These factors make it challenging to perform a global quantitative analysis of the system’s dynamics. We have observed how the coarse-grain model for the mid-temporal scale level reduced the infinite dimensional problem in real space to a limited-dimension phase-space problem. This approach can provide improved controllability, reduce variations from uncertainties, and enable quantitative analysis of the key features.

**Figure 6:**
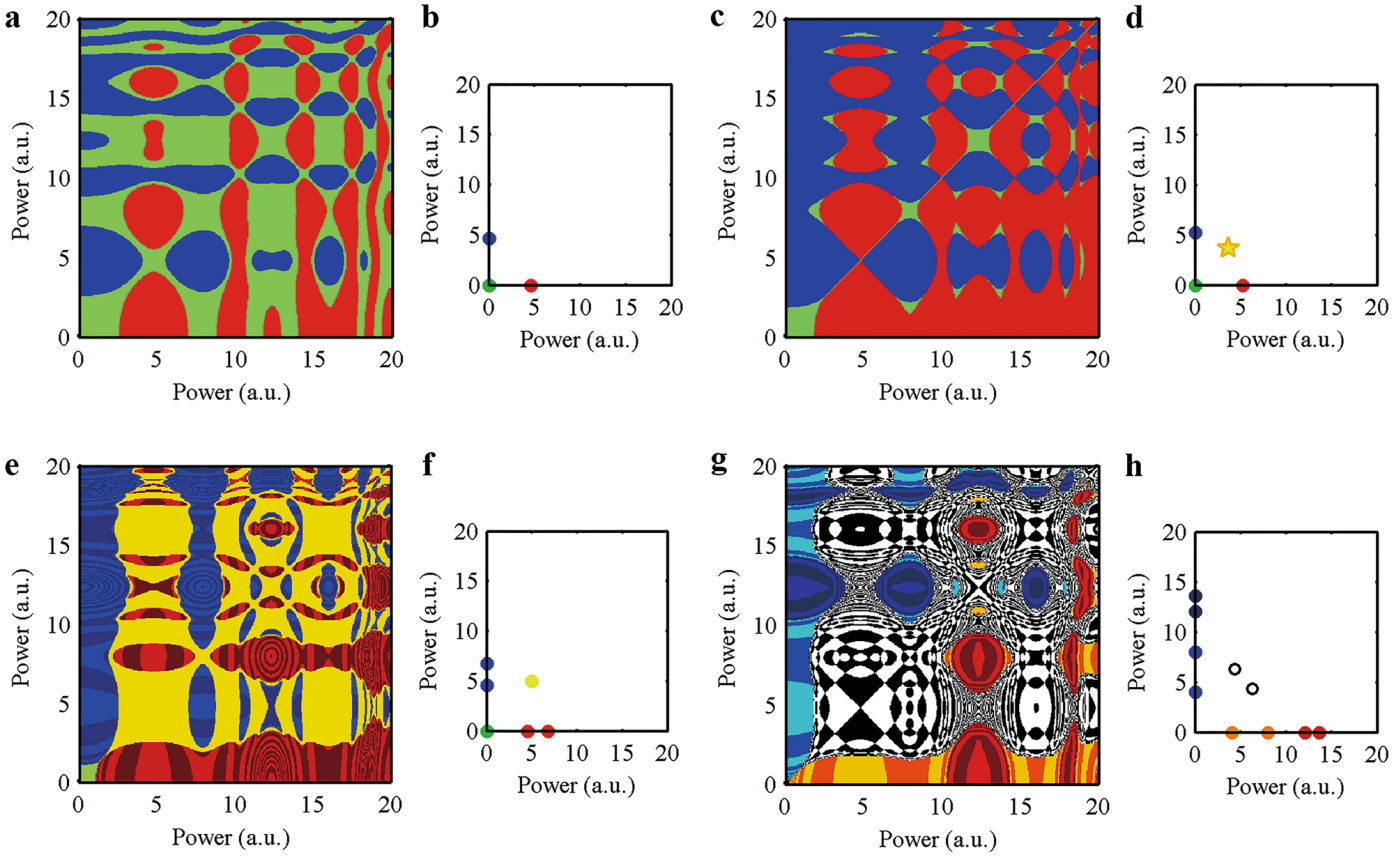
The attractors and attractor-basin phase portraits for different gain coefficients. Any initial condition is a point in phase space. A square region for possible initial condition in phase space is subdivided into 500 × 500 cells. We perform the iteration and track the points on the grid until the attractors are obtained. Then, we can derive the attractor basin. Different colours correspond to different attractors. (**a**,**c**,**e** and **g**) are attractor-basin phase portraits for different gain coefficients (g_0_); (**b**,**d**,**f** and **h**) are attractors for (**a**,**c**,**e** and **g**). For Fig. 6. (**a** and **b**), g_0_ = 1.7. There are three attractors in (**b**) (blue, red and green points). The blue point and the red point are stable single-pulse state. The green point indicates no pulse. The attractor basins for the attractors are shown in (**a**) (use the same colour as the corresponding attractor in (**b**)) For Fig. 6. (**c** and **d**), g_0_ = 2.0. There are four attractors in (**d**) (blue, red, green and yellow points). The blue point and the red point are stable single-pulse states. The green point indicates no pulse. A new attractor (the point with yellow star marker) means that the laser can support 2 pulses under a narrow range of initial conditions. For Fig. 6. (**e** and **f**), g_0_ = 2.5; There are four attractors in (**f**) (blue, red, green and yellow points). The blue points and the red points are single-pulse in periodic fluctuation state. The green point indicates no pulse. The yellow point indicates stable double pulses. For Fig. 6. (**g** and **h**), g_0_ = 3.0; There are three attractors in (**h**) (blue & dark blue, orange & red, black circle). The blue & dark blue points and the orange & red points are single-pulses in a periodic fluctuation state. The black circle is for double pulses in a periodic fluctuation state.

Tracking the evolution of all points in phase space under a given set of parameters mathematically corresponds to the attraction basin under different control parameters. The attraction basin phase portraits enable a glimpse of the entire picture and provide clues about the origin of the nonlinear phenomena in mode-locked lasers. Figure 6 and Supplementary Fig. S4 show the attractors and attractor-basin phase portraits for different gain coefficients (g_0_). By analysing this series of pictures, we can recognize patterns and then identify the source of stochastic phenomena, hysteresis and multistability. In Fig. 6(a,b), the gain coefficient (g_0_) is 1.70. There are 2 types of attractors: 1) a fixed point at the coordinate origin (i.e., no pulse); and 2) a fixed point on the x-axis or on the y-axis. In the latter case, there is a stable single pulse in the laser cavity. Figure 6(a) shows the corresponding attractor basin, where the green area in Fig. 6(a) shows the attractor basin for an attractor at the coordinate origin. If the initial state point falls in this region, then the laser cannot sustain a pulse. The blue and red areas are the respective attractor basins for the other attractors. These basins have axially symmetric shapes about the symmetry axis y = x. In this case, the laser can support only one pulse; the pulse with highest initial power predominates over the other pulses.

Figure 6(c) (g_0_ = 2.0) is similar to Fig. 6(a); however, some changes have occurred in the basins of attraction as a result of the different gain coefficient; note that the green areas are significantly reduced, and the red and blue areas are closer. We can observe the emergence of a small yellow area, which is the attractor basin for a new attractor (the point with the star marker in Fig. 6(d)). In other words, the laser can support two pulses under a narrow range of initial conditions. In Fig. 6(e,f) the gain coefficient (g_0_) is 2.5. Relative to Fig. 6(c,d) (g_0_ = 2.0), the fixed point attractor on the x-axis or y-axis is transformed into a periodic orbit, which represents a single pulse with periodic fluctuation. Smaller green areas and larger yellow areas indicate that the laser is easier to start up and more likely to enter the double pulse state than the laser in Fig. 6(c,d).

In Fig. 6(g,h) (and Supplementary Fig. S4(a,b)) the gain coefficient (g_0_) is 3.0. Periodic fluctuations appear in both the single pulse and double pulse states. The area for the fixed point attractor basin at the origin has now almost disappeared, i.e., the laser features favourable self-starting characteristics. Finally, in Supplementary Fig. S4(c,d), g_0_ = 4.5 and the attractors become chaotic attractors. The power fluctuations are relatively large under this condition.

Many reports have described the nonlinear behaviour in the laser state conversion process (stochastic behaviour, hysteresis and multistability)^28–31^. To understand the underlying mechanisms of these nonlinear phenomena, we perform an in-depth study of the attractor-basin phase portraits.

Stochastic phenomena in mode-locked lasers indicate that the same system under the same control parameters can enter different states stochastically. The pattern of the attractor basin indicates the relationship between the system’s initial state and its final state. Commonly, there are some coexisting attractors, as described above. In this case, even under the same control parameter, the system will enter a different final state if the system has an initial value that belongs to a different attractor basin. Thus, uncertainty in the initial state can cause the system to exhibit stochastic characteristics. For the mode-locked fiber laser system, stochastic phenomena commonly emerge in three cases: the start-up process, the pulse-splitting process, and the parameter-switching process. In the first two cases, ASE noise and unstable broken pulses give the system a stochastic initial position in some region of the phase space. In the third case, the system starts in either a periodic orbit or a strange attractor. The position of the point representing the state of the system changes with time in phase space. The trajectory of the point may cross multiple attractor basins for the new parameter value to which the system will be switched. Thus, the state in which the laser will finally settle is determined by two factors: the initial state at the moment the system was switched to the new parameter value, and the attractor basins for the new parameter value.

Thus, if we know the statistical properties of the initial conditions (statistical properties of the initial phase point in the phase space) and the distribution of the system’s attractor basin, we can determine the probability of a given operating state into which the system will settle.

**Figure 7:**
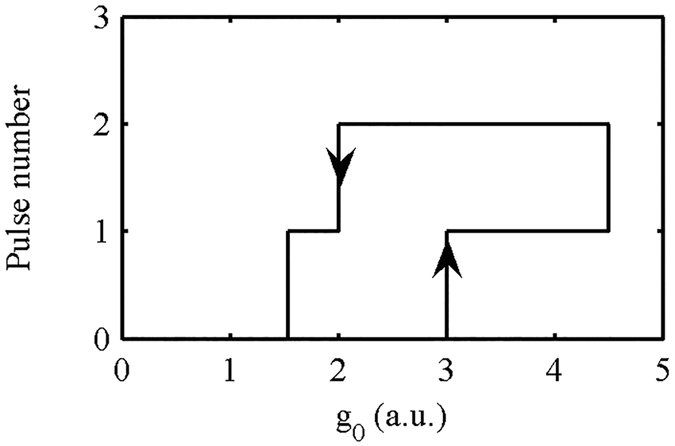
Hysteresis and multistability for pulse number in the mode-locked laser.

The coexistence of multi-attractors and their attractor basins is also the origin of hysteresis and multistability. Consider Fig. 6 as an example. We change the system status by adjusting the gain coefficient g_0_ as a control parameter. To ensure that the mode-locked laser has self-starting capability, we must increase g_0_ to a high level. Figure 6 shows that increasing g_0_ can cause expansion of the attractor basins for the single-pulse (and double pulse) status and the shrinking of the attractor basins for the 0-pulse state. This can enable the system to enter the single-pulse state (red and blue area in Fig. 6(g)) from a low power noise initial state. If g_0_ is increased to an even higher level, then we can increase the area of the attractor basins for double pulse status and get 2 pulses in the laser cavity (Supplementary Fig. S4). To change the double pulse state to a single-pulse state, we should decrease g_0_ to reduce the attractor basins for double pulses (Fig. 6(c)). If we want to extinguish the pulse in the laser, g_0_ should be decreased to a lower level than that of Fig. 6(a). This hysteresis and multistability process is shown in Fig. 7. In experiments, we often use a high gain to make the laser start up and then reduce the gain to obtain a stable single pulse. This has been verified here.

The variation of the attractor basins with the control parameter plays a crucial role in the onset of hysteresis and multistability. The mechanics of the process are as follows: attractors locate in attractor basins, and fluctuation causes the phase point that corresponds to the system state to move stochastically in the neighbourhood of the attractor in phase space. The conversion between the system states is represented by the switching between different attractors. During the course of this switching, the phase point must exit the current attractor basin and jump (drop) into a new attractor basin. To allow the system to escape from an attractor basin we can change the pattern of attractor basins by adjusting the control parameter. Shrinking or even eliminating the basin of the current attractor together with the expansion of the adjacent attractor basin can induce state conversion of the laser. When the conversion is complete, the system phase point must enter a new attractor basin with a large area. If we want to change the system state again, we can adjust the control parameter to decrease the area of this new attractor basin. On the macroscopic scale, the accumulation of control parameter changes results in a sudden change in the system state. To predict the system’s future state, both the control parameter and the history of the system must be known.

Briefly, the critical value of the state transition is jointly determined by the magnitude of the fluctuations and the shape and area of the attractor basin. Decreases in the local area of the attractor basin and increases in the fluctuations facilitate the state transition. The coexistence of fluctuations and the multi attractors is the fundamental origin of multistability and hysteresis.The macroscopic dynamic behaviour of a laser reflects the working state of the laser (single-pulse or multi-pulse, and whether it is stable). When designing a laser with a high power pulse, we often specify that the laser should operate in the stable single-pulse state, i.e., we intend for the attractor describing the laser state to be a one-dimensional fixed point. In the process of parameter optimization, we can obtain the working range and stability of the attractor in the one-dimensional fixed point state via the calculation of the attractor state and the attractor basin for the given parameters (The size of the attractor basin reflects the stability of the working state; the variation of attractor basin with the gain parameter can help us determine the laser’s operating range). Thus, we can achieve a global understanding of the characteristics of the laser with the help of our model. This can guide us to select the appropriate parameters. In addition, the study of the phenomena of hysteresis and multistability has potential importance for optical storage.

## Outlook and discussions

We have proposed the multi-level hierarchy model for mode-locked lasers and discussed it in detail for the mid-scale level (Fig. 2). Note that for the actual system, there are interactions between the various levels, where these interactions constitute the coupling channels between levels. Because of the limited space in this paper, we will indicate only the sources of these coupling channels and omit in-depth discussion. These sources are as follows.

The first source is the character of the “grain” at mid-level, i.e., the relationship between the effective peak power and the effective pulse duration (for example, for DSR, the pulse duration increases while the amplitude remains almost unchanged). (In this article, we provide the simplest example of the “grain”. Indeed, many factors, including dispersion, nonlinearity and high-order dispersion, determine the grain’s characteristics. These characteristics can be obtained by the traditional theory to provide the particle characteristic parameters for our model to analyse the macro behaviours.).

The second source is the threshold of pulse splitting and the property of the new pulses that emerge after pulse splitting.

The third source involves large time-scale processes, such as Q-switching, in which case the transient characteristics of the rare-earth doped fiber should be considered. Statistical properties and pulse envelopes can be used for simplification.

Note that the “grains” (pulses) in the laser cavity we study here are relatively independent from each other, although the gain saturation of the amplifier can cause the indirect interaction between the pulses^13, 14, 16^. In some cases, there is a kind of soliton pulse –the bisoliton can be made using a mode-locked laser with special design in experiment^56^. In this case, two (or even more) consecutive dissipative solitons preserve a small equilibrium distance between them. The two pulses have overlapped optical fields in the time domain, and they can interact with each other directly^56^. In this situation, the bisoliton cannot be treated as two independent pulses. It is necessary to consider the soliton-soliton interaction when analysing such pulses. The bisoliton should be studied as a special “grain” or “coupled grain pair” instead of two independent “grains” at the mid-level of the hierarchical model. The characteristics of this kind of special “grain” should be analysed by the bottom-level model considering the action between the pulses. Some useful discussion about the bisoliton can be found in ref. 56.

In the previous discussion, the amplifier gain model (Eq. (4)), which is widely used for the analysis of dynamic characteristics of mode-locked fiber lasers (e.g. refs 14–16, 28, 33, 57 and 58), is a simplified model. When we consider the dynamics of the mode-locked fiber laser at large time scales together with the temporal response of the erbium doped fiber amplifier (EDFA), a complete EDFA transient model with the time-dependent rate equation^59, 60^ should be used.

6 $$\frac{d{n}_{2}}{dt}=\sum _{k}\frac{{P}_{k}{i}_{k}{\sigma }_{ak}}{h{\nu }_{k}}{n}_{2}(r,\varphi ,z)-\sum _{k}\frac{{P}_{k}{i}_{k}{\sigma }_{ek}}{h{\nu }_{k}}{n}_{1}(r,\varphi ,z)-\frac{{n}_{2}(r,\varphi ,z)}{\tau }$$

The model contains many parameters of an EDFA (doping, absorption coefficient, emission coefficient, pump, etc.). The model can describe the interaction of the pulse and an EDFA more precisely.

For the EDFA in the mode-locked laser system, a train of ultra-short pulses with high peak power is injected into it. When the high peak power pulse arrives, the inversion level decreases to a low level immediately. (Mathematically, the step change is obtained by integrating the impulse signal.) Next, the reverse level rises gradually by the pump power injected into the EDFA until the next pulse comes. The recovery process of the inversion level is determined by the specific characteristics of the EDFA and the pump. When the time interval between pulses in the mode-locked fiber laser is small, (particularly in the case of multi-pulse mode-locking status), the inversion level of the EDFA is always in the process of dynamic fluctuation. The fluctuation is related to the magnitude of the input pulses and the characteristics of the EDFA.

This model can be used in studying large time-scale pulse fluctuations and Q-switch mode locking in which the EDFA’s transient dynamics are involved.

In addition, note that for the model discussed in this article, the pulses are assumed to have some special and stable shapes (e.g., generalized solitons). For pulse waveforms without a stable shape (e.g., rogue waves ref. 61), a separate discussion is required.

From the perspective of dissipative system theory, the mode-locked fiber laser is a thermodynamically open system operating far from thermodynamic equilibrium in an environment with which it exchanges energy^47–49^. The laser excitation occurs when the energy supply exceeds the threshold. For pulsed excitation, the mode locker is the key component for symmetry breaking in the time domain. The symmetry breaking together with the joint action of saturation and feedback cause the emergence of temporal patterns. Under the action of self-organization we can see that a system can vary from spontaneous emission in the disordered state to a continuous wave laser with a highly ordered state in the frequency domain, then to a pulsed laser with a highly ordered state in time domain (pulse with a specific shape Supplementary Video S9) and finally to a laser with multiple pulses that shows ordered and chaotic macro behaviour (Supplementary Videos S8–S10). Furthermore, the analysis of the attractors’ basin tells us that the fluctuations, the coexistence of attractors, and the variation of the attractors’ basins are the driving forces for the evolution and macroscopic nonlinear behaviour of the system.

We have proposed a new method that can clarify the chaotic behaviour and help explain the complexities of the behaviour exhibited by mode-locked lasers. At the methodological level, the proposed method may have a wide range of applications in the fields of physics, nonlinear dynamics and complex systems.

## Methods

### The direct numerical model and parameters for mode-locked laser

The mode-locked laser is composed of optical fibers (gain fiber and standard single-mode fiber (SMF) and components (mode locker and output coupler)). The model is based on simulating every part of the oscillator (Fig. 1) separately.

In the fibers numerical simulations are based on a modified nonlinear Schrödinger equation^6, 8^ which can be solved by the split-step Fourier method^35^.

7 $$\frac{\partial U}{\partial z}-\frac{j}{2}{\beta }_{2}\frac{{\partial }^{2}U}{\partial {\tau }^{2}}-\frac{1}{6}{\beta }_{3}\frac{{\partial }^{3}U}{\partial {\tau }^{3}}+\frac{\alpha }{2}U=\frac{g}{2}U-j\gamma {|U|}^{2}U-j\nu {|U|}^{4}U$$

Here, *U*(*z*, *τ*) is the slowly varying amplitude of the pulse envelope, *z* is the propagation coordinate, and τ is the retarded time. *α* is the attenuation constant. *β*_2_ and *β*_3_ are the second-order (GVD) and the third-order dispersion (TOD) parameters, respectively. *γ* is the cubic nonlinearity. *v* accounts for the quintic nonlinearity^8^.

The gain fiber has the following gain: g = g_0_/[1 + E_pulse_/E_sat_ ].

g_0_ is the small-signal gain. E_pulse_ is the energy of all the pulses in the cavity; E_sat_ is the gain saturation energy. Additionally, the gain spectrum is simplified to a parabolic shape with a gain bandwidth of 40 nm.

Note that because the nonlinear loss model is used for the mode locker, the scalar equation (Eq. (7)) can be used. If the nonlinear polarization rotation model for the model locker is given and the nonlinear loss is unknown, then the coupled complex nonlinear Schrödinger equations^57, 62^ should be used.

**The parameters for** **Fig.** **4(e,f)** **:**

Erbium doped fiber (EDF): The EDF has a length of 10 m, g_0_ = 2.38 dB/m, α = 0, β_2_ = 10 ps^2^/km, β_3_ = 0, γ = 5.0 (km^−1^W^−1^), E_sat_ = 50 (pJ).

Single-mode fiber (SMF): The SMF has a length of 11 m, g_0_ = 0, α = 0.17 dB/km, β_2_ = −23.6 ps^2^/km, β_3_ = 0, γ = 1.387 (km^−1^W^−1^), ν = 0 (km^−1^W^−3^).

The coupling ratio of the coupler is 40:60 (60% of the power is extracted from the cavity).

The mode locker is modelled by a nonlinear loss function (periodic nonlinear loss for nonlinear polarization rotation based laser)^16, 42^.

8 $${\rm{Loss}}=1-\{{{\rm{M}}}_{0}+{{\rm{M}}}_{{\rm{N}}}\cdot [1-\,\cos (2{\rm{\pi }}\cdot \frac{{\rm{P}}-{{\rm{P}}}_{{\rm{\theta }}}}{{{\rm{P}}}_{{\rm{M}}}})]\}$$

$${{\rm{M}}}_{{\rm{0}}}=0.3,{{\rm{M}}}_{{\rm{N}}}=0.2,{{\rm{P}}}_{{\rm{M}}}=32,\,and\,{{\rm{P}}}_{{\rm{\theta }}}=0.$$

Here we use Eq. (8) to model the nonlinear loss for mode locking using nonlinear polarization rotation. (In this model, the nonlinear loss curve is known to generate a periodic structure at higher intensities^16, 33, 42, 45^). Because the nonlinear loss is directly related to the nonlinear dynamical behaviour of the laser, the model can be conveniently used for dynamic analysis.

Note that another model is often used for the nonlinear polarization rotation mode-locked fiber laser. (The model has been well described in refs 57 and 62). The model is a direct simulation of the physical process of the polarization rotation in the laser cavity. Unlike the nonlinear loss model, the advantage of this model is that it has a stronger correlation with the parameters and characteristics of the device (e.g., the fiber, the polarization controllers, and the angle of the polarizer and analyser). Thus, it can be more easily associated with the experiment. However, the relationship between the parameters and the final nonlinear loss curve is indirect.

In the actual laser design process, first, the nonlinear loss model can be used to analyse and design the laser to obtain the optimized mode-locker loss curve. Next, the specific parameters of the device are set by reverse engineering and using the model based on physical process simulation. This method has been used to optimize the multiple transmission filters for nonlinear polarization rotation mode-locked fiber laser^42, 45^ and to obtain the DSR in the polarization rotation mode-locked fiber lasers^43^.

### The coarse-grain model and parameters for mode-locked lasers

The equations and the introduction are detailed in the main text (Eqs (1–5)). For the schematic diagram, see Fig. 1(c,d).

The pulse duration is irrelevant to pulse energy and is normalized to be 1 (Eq. (2) becomes: t_eff_ = 1).

The mode locker is modelled by a nonlinear loss function (periodic nonlinear loss for nonlinear polarization rotation based laser)^16, 42^.

**The parameters for** **Fig.** **3** **:**

M_0_ = 0.1, M_N_ = 0.36, P_M_ = 8, P_θ_ = 0 (Eq. (8)); E_sat_ = 5 (Eq. (4)).

The cavity loss caused by the coupler is 50% (50% of the power is extracted from the cavity).

The initial condition: x_1_ = 3.00, x_2_ = 2.12 (Eq. (1) in the main text).

### Method for obtaining the attractors

To obtain the attractors (e.g., Fig. 4(a,b,c,d)), we iterate for 1000 cycles to cause the transient effects of the system to decay. After the system settles down to its eventual state, we then plot points for the following 10,000 iterations in phase space.

**The parameters for** **Fig.** **4(a,b,c,d)** **:**

M_0_ = 0.1, M_N_ = 0.3 (M_N_ = 0.1 for Fig. 4(b)), P_M_ = 8, P_θ_ = 0 (Eq. (8)); E_sat_ = 5 (Eq. (4));

Fig. 4(a) The initial condition: x_1_ = 3.00, x_2_ = 2.12; the gain coefficient: g_0_ = 3.2;

Fig. 4(b) The initial condition: x_1_ = 5.00, x_2_ = 1.00; the gain coefficient: g_0_ = 7.0;

Fig. 4(c) The initial condition: x_1_ = 3.00, x_2_ = 2.12, x_3_ = 2.00; the gain coefficient: g_0_ = 3.90;

Fig. 4(d) The initial condition: x_1_ = 3.00, x_2_ = 2.12, x_3_ = 2.00; the gain coefficient: g_0_ = 3.95.

### Method to getting the attractor basin

Any initial condition is nothing more than a point in phase space. A square region for the possible initial condition in phase space is subdivided into 500 × 500 cells. We perform the iteration and trace the points on the grid until we obtain the attractors. Then, we can derive the attractor basin. Different colours correspond to different attractors.

**The parameters for** **Fig.** **5** **:**

M_0_ = 0.1, M_N_ = 0.36, P_M_ = 8, P_θ_ = 0 (Eq. (8)); E_sat_ = 5, g_0_ = 2.45 (Eq. (4)).

The cavity loss caused by the coupler is 50% (50% of the power is extracted from the cavity).

The initial condition: x_1_ = 3.00 (Eq. (1)).

**The parameters for** **Fig.** **6** **:**

M_0_ = 0.1, M_N_ = 0.3, P_M_ = 8, P_θ_ = 0 (Eq. (8)); E_sat_ = 5 (Eq. (4)).

The cavity loss caused by the coupler is 50% (50% of the power is extracted from the cavity).

The gain coefficient: g_0_;

For Fig. 6(a,b) g_0_ = 1.7;

For Fig. 6(c,d) g_0_ = 2.0;

For Fig. 6(e,f) g_0_ = 2.5;

For Fig. 6(g,h) g_0_ = 3.0.

**The parameters for** **Fig.** **7** **:**

M_0_ = 0.1, M_N_ = 0.3, P_M_ = 8, P_θ_ = 0 (Eq. (8)); E_sat_ = 5 (Eq. (4)).

## Electronic supplementary material

Supplementary information

Video S1

Video S2

Video S3

Video S4

Video S5

Video S6

Video S7

Video S8

Video S9

Video S10
