# Supplementary material for: General description and understanding of the nonlinear dynamics of mode-locked fiber lasers
Source: Sci Rep. 2017 May 2;7:1292. doi: 10.1038/s41598-017-01334-x (PMC5431068; doi:10.1038/s41598-017-01334-x)
Supplement: Supplementary file 1 — Supplementary information [file 41598_2017_1334_MOESM1_ESM.doc]

**Supplementary information**

***General description and understanding of the nonlinear dynamics of mode-locked fiber lasers***

Huai Wei*, Bin Li, Wei Shi*, Xiushan Zhu, Robert A. Norwood, Nasser Peyghambarian, and Shuisheng Jian

CONTENTS

**1. Bifurcation diagram and Liapunov exponent 1**

Video S1 1

Video S2 2

Figure S1 3

**2. Attractor and Correlation dimension 4**

Video S3 4

Video S4 - S7 5

Figure S2, S3 7

Table S1 8

**3. Attractor Basin 9**

Figure S4 9

**4. Self organization of pulse evolution in mode locked laser 10**

Video S8 10

Video S9 11

Video S10 12

**1. Bifurcation diagram and Liapunov exponent**


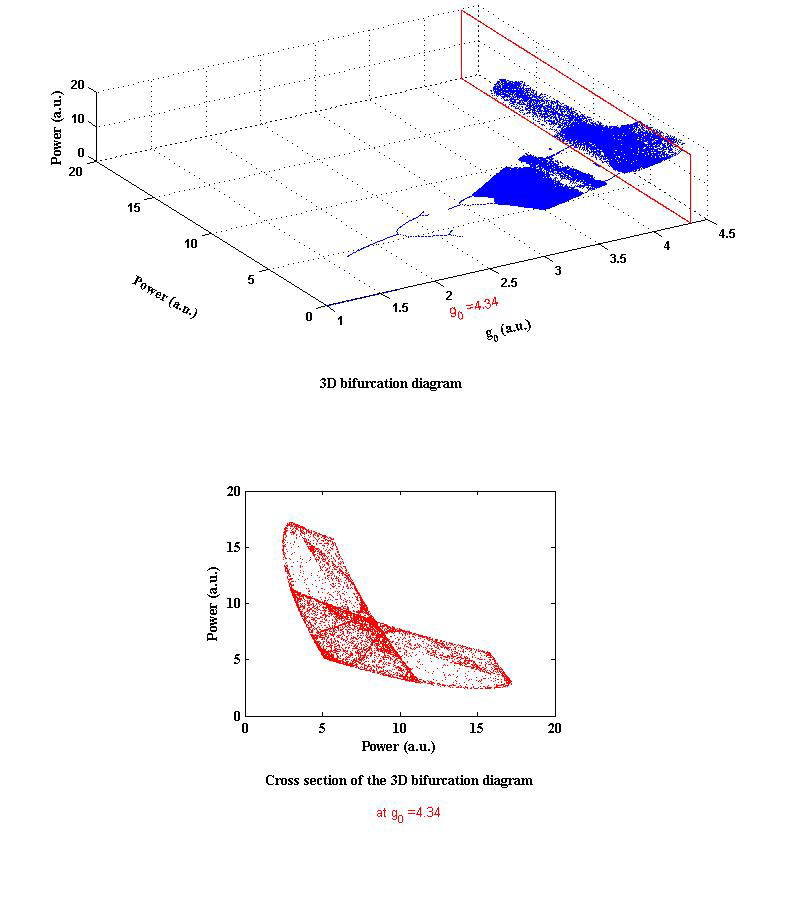


**Video S1.** Bifurcation diagram and the attractor for a the given gain coefficient (g_0_)

**(The video is provided in a separate file Video.S1.gif)**

The upper panel shows the bifurcation orbit diagram in multi-dimensional phase space under certain initial conditions with different gain coefficient (g_0_). The lower panel shows the attractor for certain gain coefficient g_0_ (the cross-section of the bifurcation diagram at g_0_).

**The parameters for Video S1:**

M_0_=0.1, M_N_=0.3, P_M_=8, P_θ_=0 (Equation (8) in the main text); E_sat_=5 (Equation (4) in the main text);

Gain coefficient: g_0_

The cavity loss caused by the coupler is 50% (50% of the power is extracted from the cavity);

The initial condition: x_1_=3.00 , x_2_=2.12 (Equation (1) in the main text ).


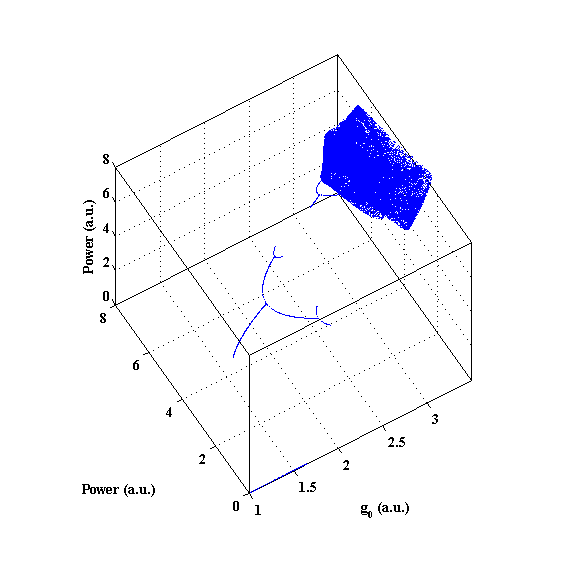


**Video S2.** 3D bifurcation diagram in multi-dimensional phase space under certain initial conditions with different gain coefficient g_0_.

**(The video is provided in a separate file Video.S2. gif)**

The parameters for Video S2 are the same as for Video S1.


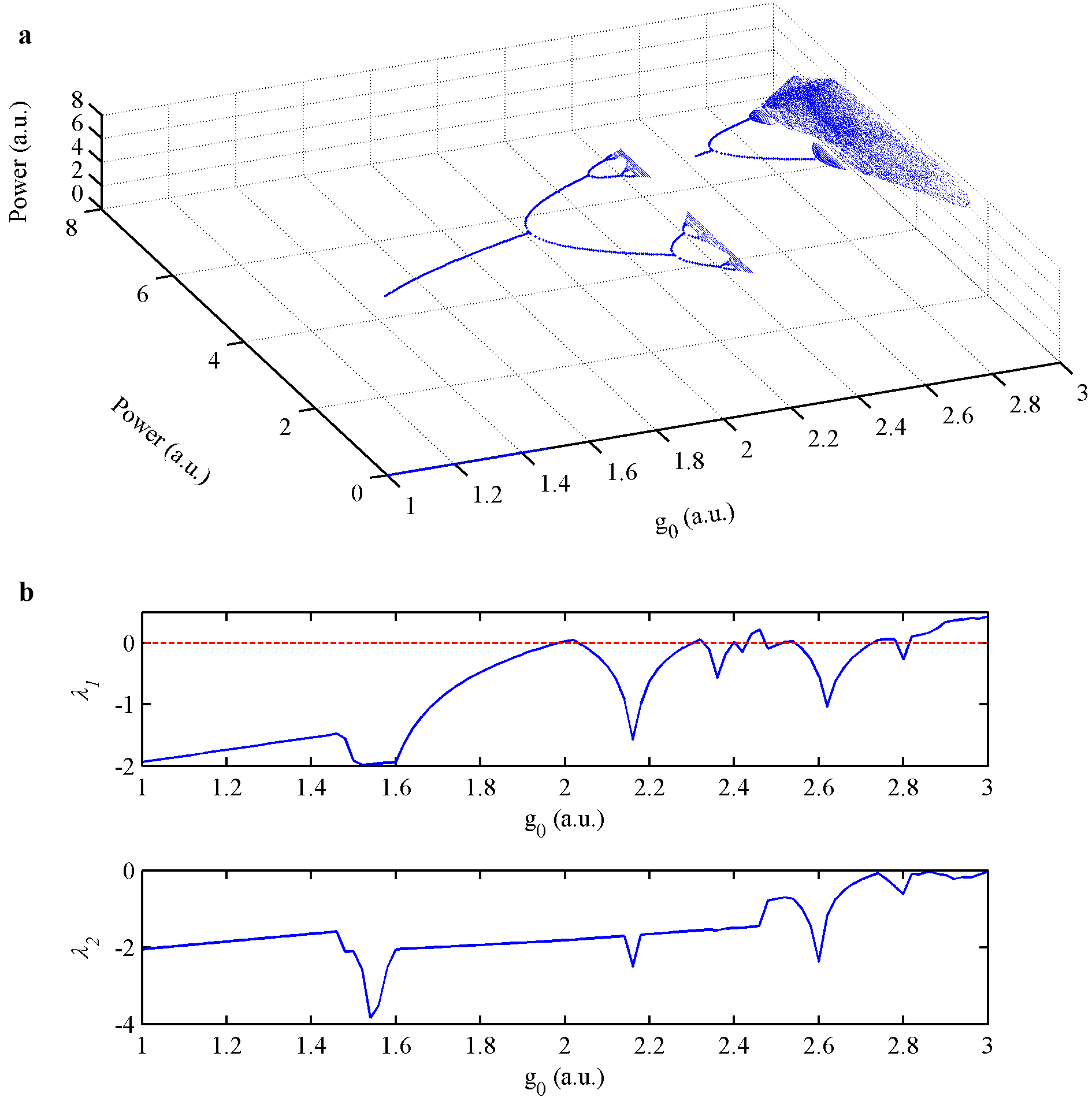


**Figure S1.** Bifurcation diagram and Lyapunov exponent

**a**, Bifurcation diagram in multi-dimensional phase space under certain initial conditions with different gain coefficient g_0_.

**b,** The first and second Lyapunov exponent (derived by Wolf’s method).

**The parameters for Figure S1:**

M_0_=0.1, M_N_=0.36, P_M_=8, P_θ_=0 (Equation (8) in the main text); E_sat_=5 (Equation (4) in the main text);

The cavity loss caused by the coupler is 50% (50% of the power is extracted from the cavity);

The initial condition : x_1_=3.00 , x_2_=2.12 (Equation (1) in the main text ).


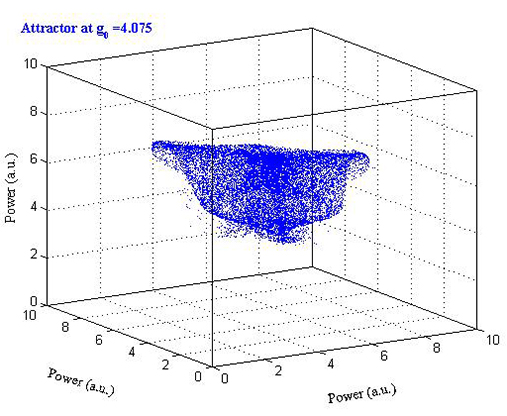
**2. Attractor**

**Video S3.** The attractor’s evolution with the variation of the gain coefficient g_0_ in the 3D phase space

**(The video is provided in a separate file: Video.S3.gif)**

**The parameters for Video S3:**

M_0_=0.1, M_N_=0.3, P_M_=8, P_θ_=0 (Equation (8) in the main text); E_sat_=5 (Equation (4) in the main text);

The cavity loss caused by the coupler is 50% (50% of the power is extracted from the cavity);

The initial condition : x_1_=6.0, x_2_=7.12, x_3_=8.0 (Equation (1) in the main text );

gain coefficient: g_0_.

**
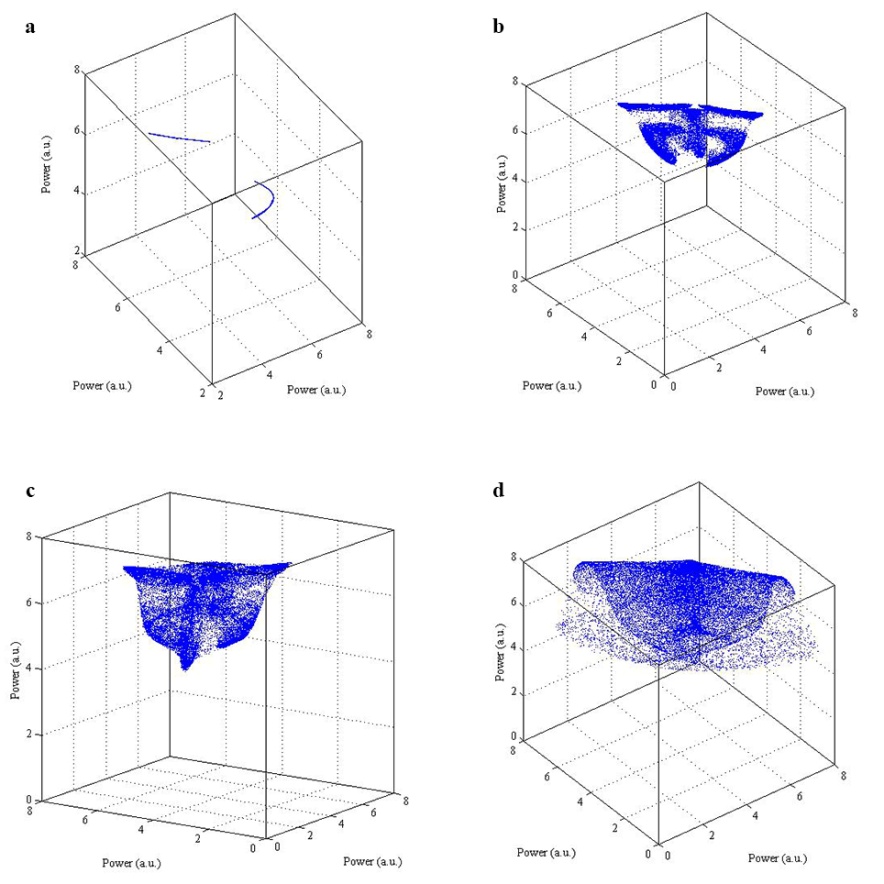
**

**Video S4 - S7.** Some attractors for three pulses in 3D phase space

a,b,c,d, respectively, video grab images of Video S4 - S7

**（The videos are provided in separate files: Video.S4-S7.gif）**

**The parameters for Video S4-S7:**

M_0_=0.1, M_N_=0.3, P_M_=8 , P_θ_=0 (Equation (8) in the main text); E_sat_=5 (Equation (4) in the main text);

The cavity loss caused by the coupler is 50% (50% of the power is extracted from the cavity);

The initial condition : x_1_=6.0, x_2_=7.12, x_3_=8.0 (Equation (1) in the main text );

The gain coefficient: g_0_

Video S4: g_0_=3.90; Video S5: g_0_=3.94; Video S6: g_0_=3.95; Video S7**:** g_0_=4.15.


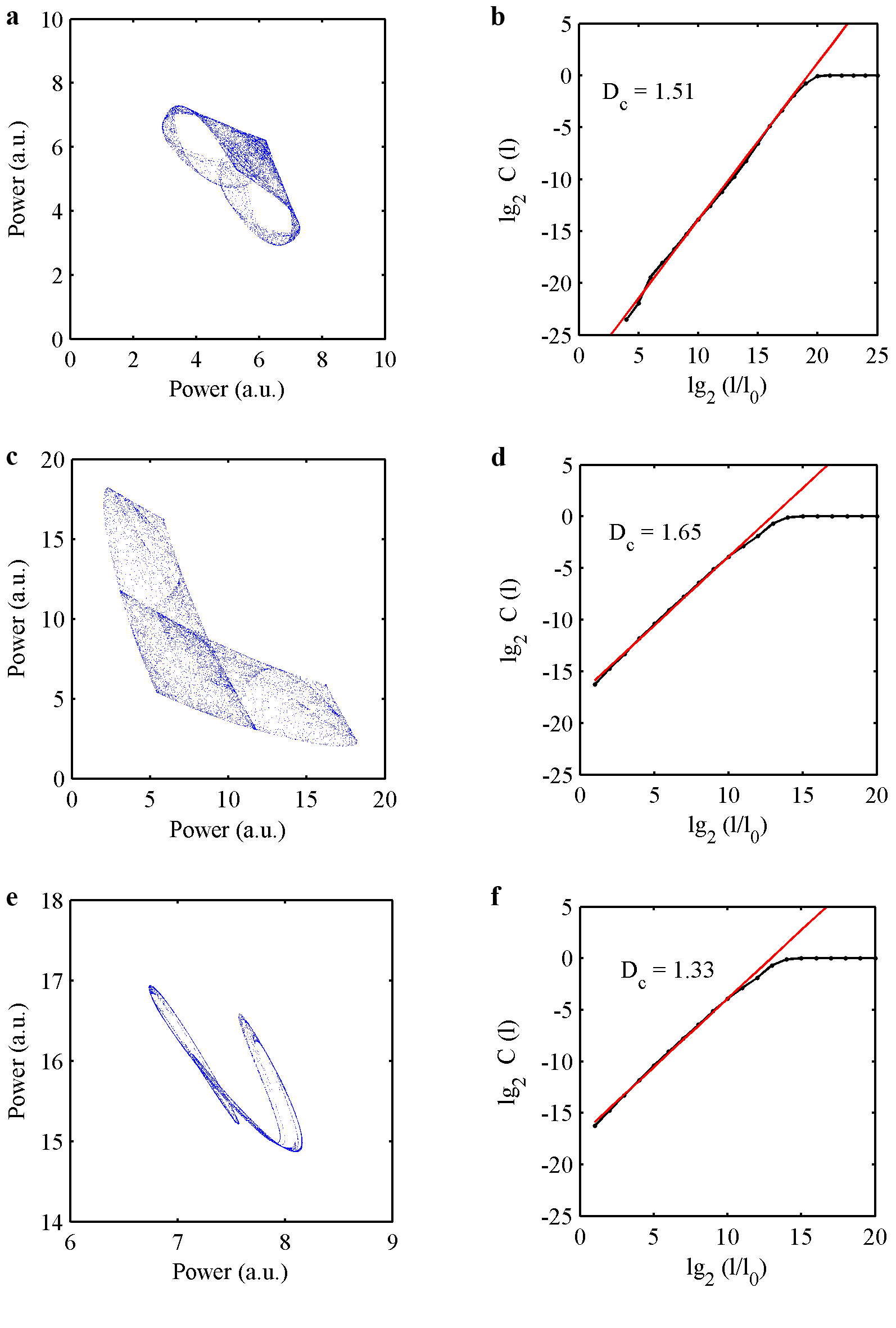


**Figure S2.** Strange attractors and correlation dimension for two pulses

**The parameters for Figure S2:**

M_0_=0.1, M_N_=0.3, P_M_=8, P_θ_=0 (Equation (8) in the main text); E_sat_=5 (Equation (4) in the main text);

The cavity loss caused by the coupler is 50% (50% of the power is extracted from the cavity);

The initial condition : x_1_=3.0, x_2_=2.12 (Equation (1) in the main text);

The gain coefficient: g_0_

g_0_ =3.2 for Figure S2 a,b; g_0_ =4.5 for Figure S2 c,d; g_0_ =7.0 for Figure S2 e,f.


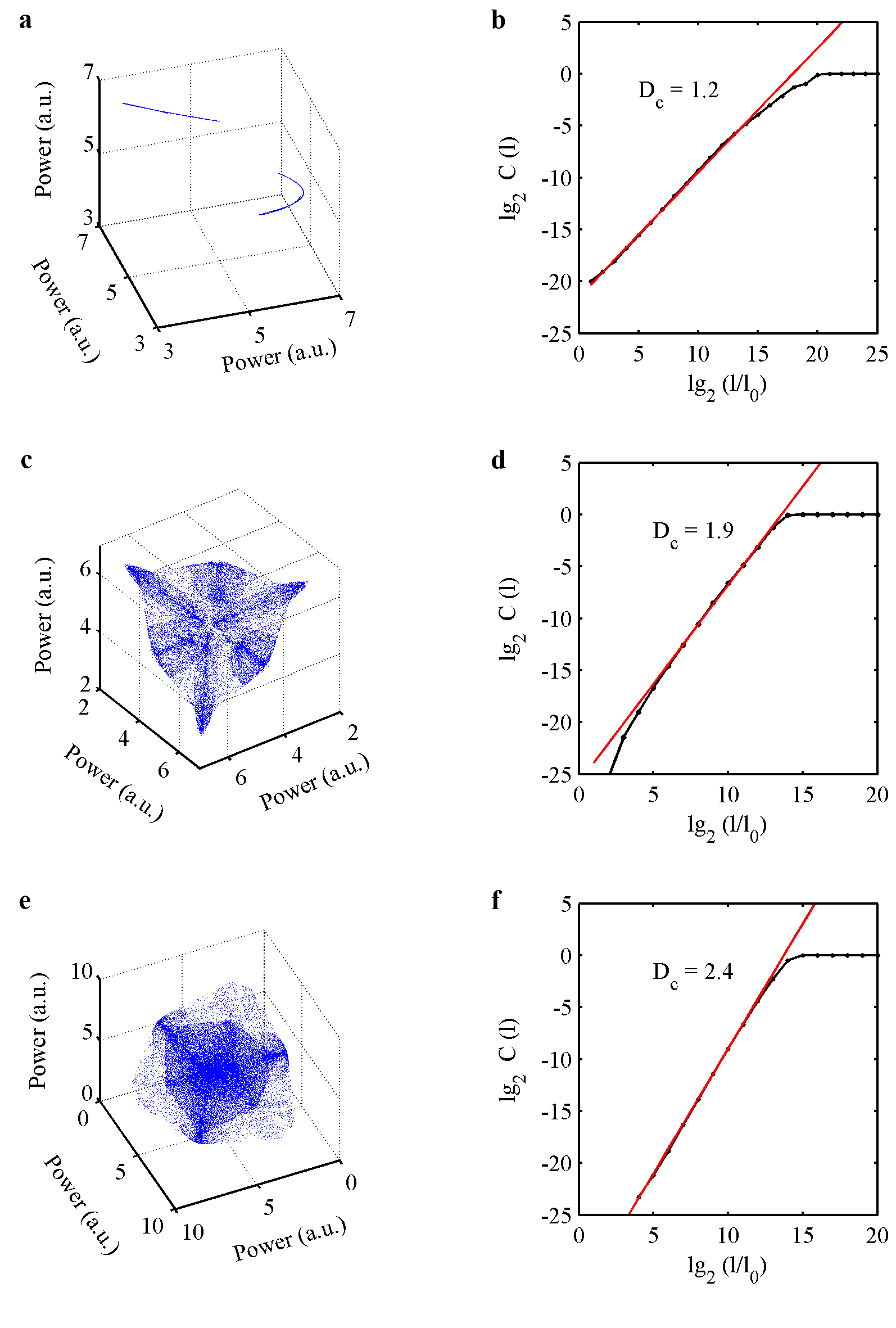


**Figure S3.** Strange attractors and correlation dimension for three pulses

**The parameters for Figure S3:**

M_0_=0.1, M_N_=0.3, P_M_=8, P_θ_=0 (Equation (8) in the main text); E_sat_=5 (Equation (4) in the main text);

The cavity loss caused by the coupler is 50% (50% of the power is extracted from the cavity);

The initial condition : x_1_=6.0 , x_2_=7.12, x_3_=8.0 (Equation (1) in the main text );

The gain coefficient: g_0_=3.90 for Figure.S3 a,b；g_0_ =3.95 for Figure.S3 c,d；g_0_ =4.15 for Figure.S3 e,f；

**Table S**1

| **Pulse number** | **Space dimension** | **Pulse state** | **The attractor and it’s dimension** | **Figure** |
| --- | --- | --- | --- | --- |
| **Single pulse** | 1 | Stable single pulse | A point in 1D space;  dimension：D=0 | Fig.3 point A |
|  | 1 | Single pulse with periodic fluctuation | Two (or more) discrete points  dimension：D=0 | Fig.3 point B,C |
|  | 1 | Single pulse with chaos fluctuation | Strange attractor in 1D space  dimension:  0<D<1 | Fig.3 point E |
| **Two pulses** | 2 | Stable two pulses | A point in 2D space;  dimension：D=0 | Fig.3 point F |
|  | 2 | Two pulse with periodic fluctuation | Two (or more) discrete points  in 2D space  dimension：D=0 | Fig.3 point G |
|  | 2 | Two pulse with chaos fluctuation | Strange attractor in 2D space  dimension :0<D<2 | Fig.3 point H, Figure S2  Fig.4 a,e,f *1 |
|  | 2 | Asymmetric two pulses | The attractor is asymmetrical about “x =y” | Fig.4 b, Figure S2.e  *2 |
| **Three pulses** | 3 | **…***3 | **…** | VideoS.4 |
|  | 3 | Three pulse with chaos fluctuation | Strange attractor in 3D space  0<D<3 | Figure.S3，Fig.4 c,d  VideoS.4-S.7  *4 |
|  |  |  |  |  |
|  |  |  |  |  |

***1, *2, *4 :** New states for pulses in mode-locked laser which have not yet reported.

***3:** Similar to the previous case (Stable, periodic fluctuation, chaos fluctuation).

**3. Attractor Basin**


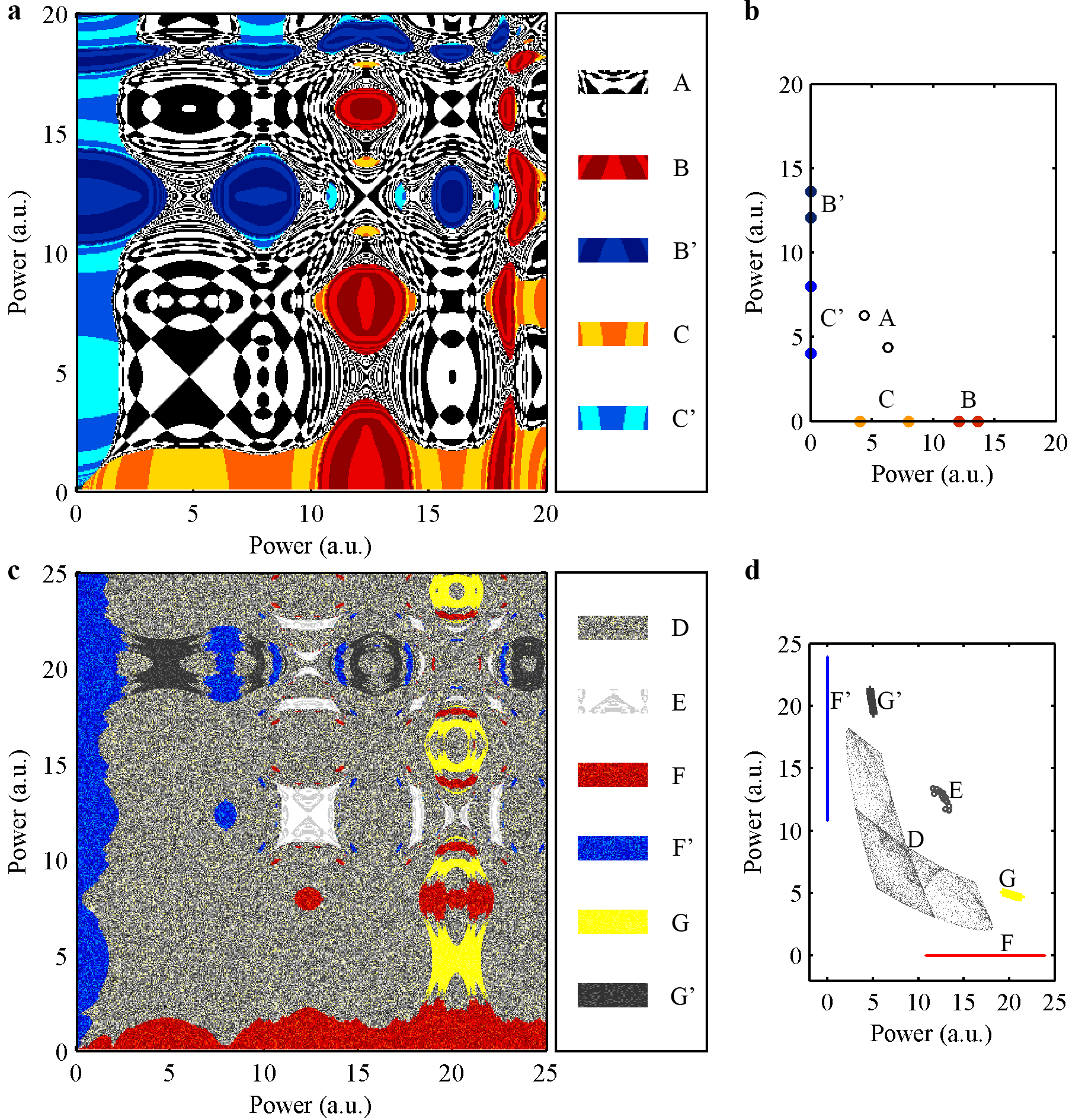


**Figure S4.** The attractors (**b, d**) and attractor-basin (**a,c**) phase portraits for different gain coefficient

**The parameters for Figure S4:**

M_0_=0.1, M_N_=0.3, P_M_=8, P_θ_=0 (Equation (8) in the main text); E_sat_=5 (Equation (4) in the main text);

The cavity loss caused by the coupler is 50% (50% of the power is extracted from the cavity);

The gain coefficient: For Fig S4. a, b, g_0_=3.0; For Fig S4. c, d, g_0_=4.5.

**4. Self organization of pulse evolution in mode locked laser**


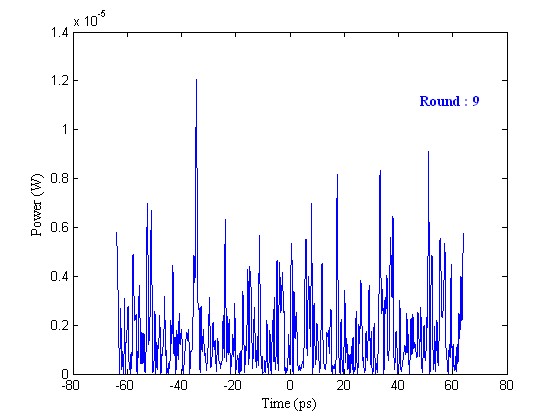

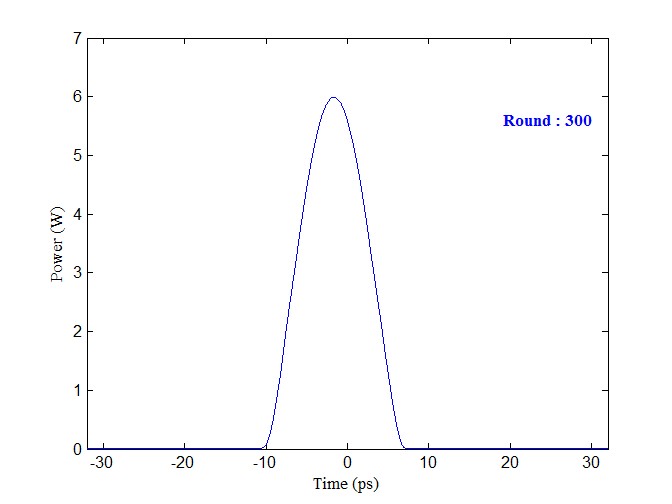


**Video S8.** The evolution from spontaneous emission in disordered state to a pulse laser with highly ordered state in time domain

**(The video is provided in a separate file*:* Video.S8.gif)**

**The parameters for Video S8:**

EDF: The EDF has a length of 6.6 m, g_0_=1.50dB/m, α=0, β_2_= 10ps^2^/km, β_3_=0, γ=5.0 (km ^-1^W^-1^) , ν=0 (km ^-1^W^-3^), E_sat_  =50 (pJ);

SMF: The SMF has a length of 1 m, g_0_=0, α=0.17dB/km, β_2_ = -23.6 ps^2^/km, β_3_=0, γ=1.387 (km^-1^W^-1^), ν=0 (km ^-1^W^-3^);

DSF: The DSF has a length of 3 m, g_0_ =0, α=0.17dB/km, β_2_= 7.0 ps^2^/km, β_3_=0, γ=5.0 (km ^-1^W^-1^), ν=0 (km ^-1^W^-3^);

The coupling ratio of the coupler is 40:60 (60% of the power is extracted from the cavity);

The nonlinear loss of the mode locker (saturable absorber) is modeled by:

Loss=1-[L_0_/(1+P/P_Msat_)],

L_0_=0.7, P_Msat_=1.


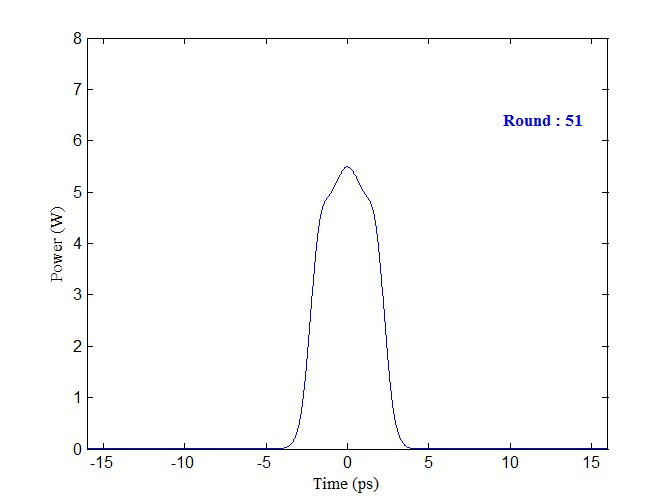


**Video S9** An Gaussian pulse evolution to be a DSR pulse automatically

**(The video is provided in a separate file: Video.S9.gif)**

**The parameters for Video S9:**

EDF: The EDF has a length of 6.6 m, g_0_=1.50dB/m, α=0, β_2_= 10ps^2^/km, β_3_ =0, γ=5.0 (km ^-1^W ^-1^), ν= -0.5 (km ^-1^W^-3^), E_sat_=50 (pJ);

SMF: The SMF has a length of 8 m, g_0_=0, α=0.17dB/km, β_2_ = -23.6 ps^2^/km, β_3_ =0, γ=1.387 (km^-1^W ^-1^), ν=0 (km ^-1^W^-3^);

DSF: The DSF has a length of 3 m, g_0_ =0, α=0.17dB/km, β_2_= 7.0 ps^2^/km, β_3_ =0, γ=5.0 (km ^-1^W ^-1^), ν=0 (km ^-1^W^-3^);

The coupling ratio of the coupler is 40:60 (60% of the power is extracted from the cavity);

The nonlinear loss of the mode locker (saturable absorber) is modeled by:

Loss=1-[L_0_/(1+P/P_Msat_)]e^-1(P/Psat )^

L_0_=0.7, P_Msat1_=1, P_sat_=250.


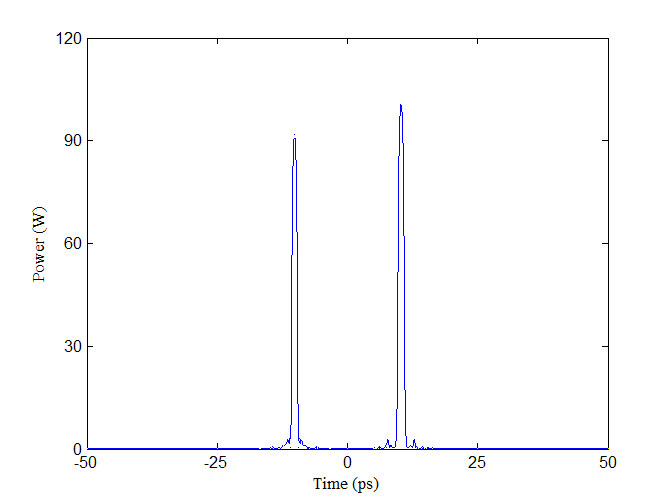


**Video.S10** Chaos state of two pulses derived by direct numerical simulation

**(The video is provided in a separate file: Video.S10.gif)**

The parameters for Video S10 are the same as the parameters for Fig. 4 e, f (see Methods)
