# Supplementary figures and images for: General description and understanding of the nonlinear dynamics of mode-locked fiber lasers
Source: Sci Rep. 2017 May 2;7:1292. doi: 10.1038/s41598-017-01334-x (PMC5431068; doi:10.1038/s41598-017-01334-x)

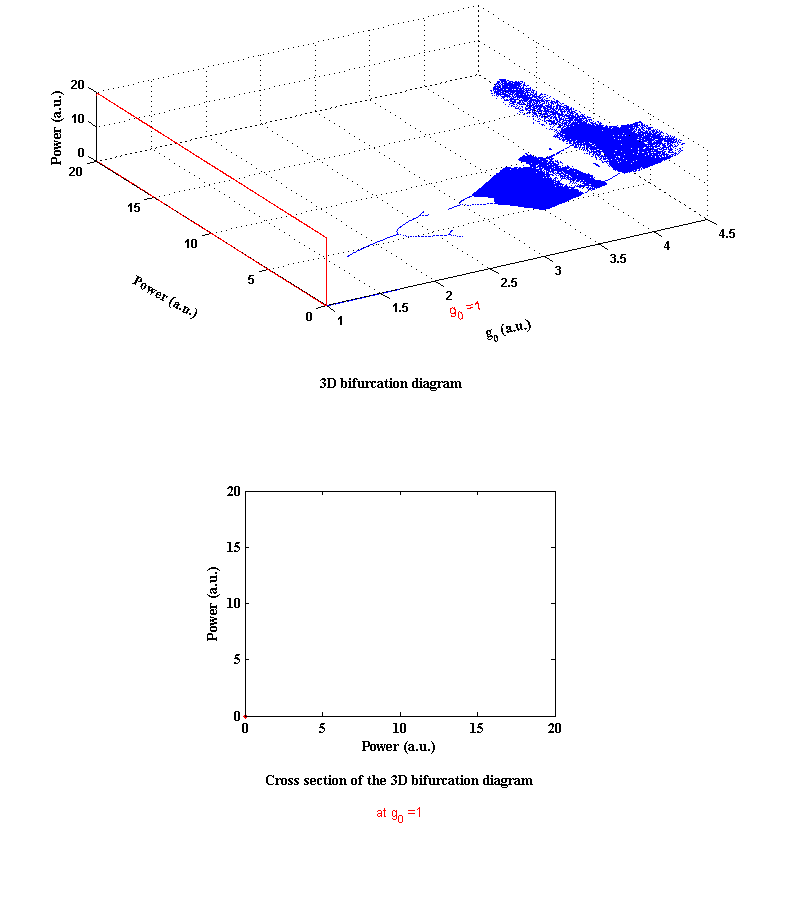

Supplement: Supplementary file 2 — Video S1 [file 41598_2017_1334_MOESM2_ESM.gif]

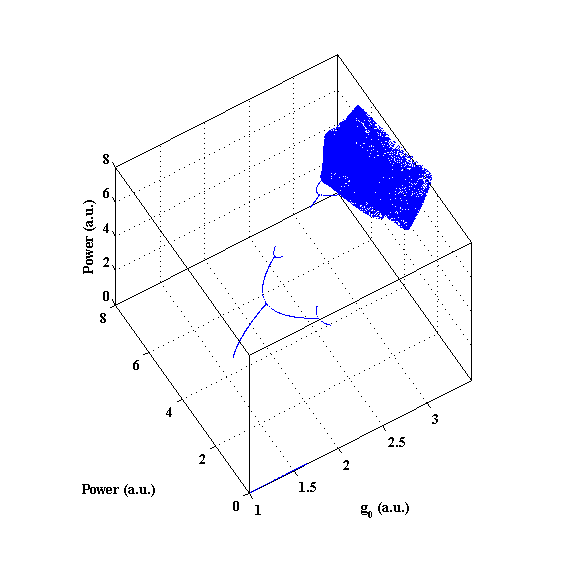

Supplement: Supplementary file 3 — Video S2 [file 41598_2017_1334_MOESM3_ESM.gif]

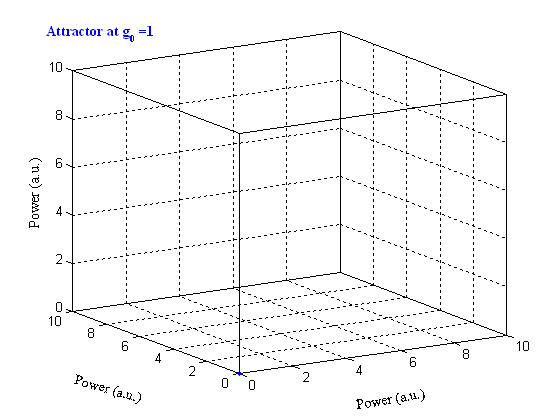

Supplement: Supplementary file 4 — Video S3 [file 41598_2017_1334_MOESM4_ESM.gif]

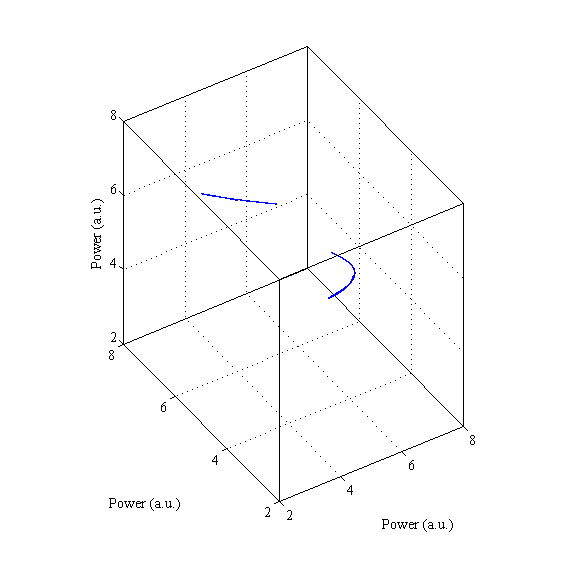

Supplement: Supplementary file 5 — Video S4 [file 41598_2017_1334_MOESM5_ESM.gif]

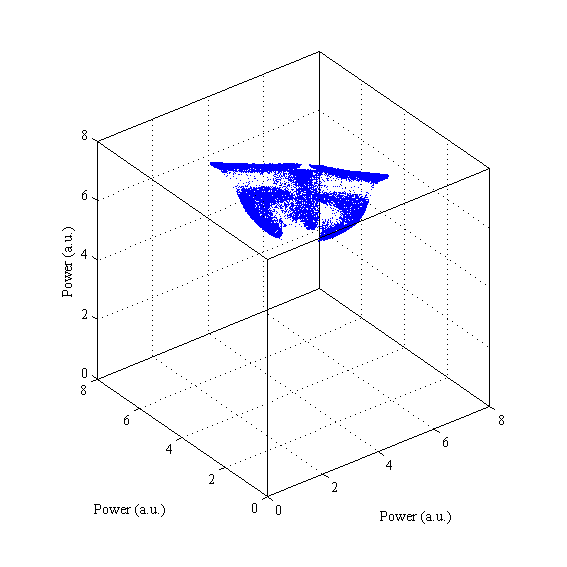

Supplement: Supplementary file 6 — Video S5 [file 41598_2017_1334_MOESM6_ESM.gif]

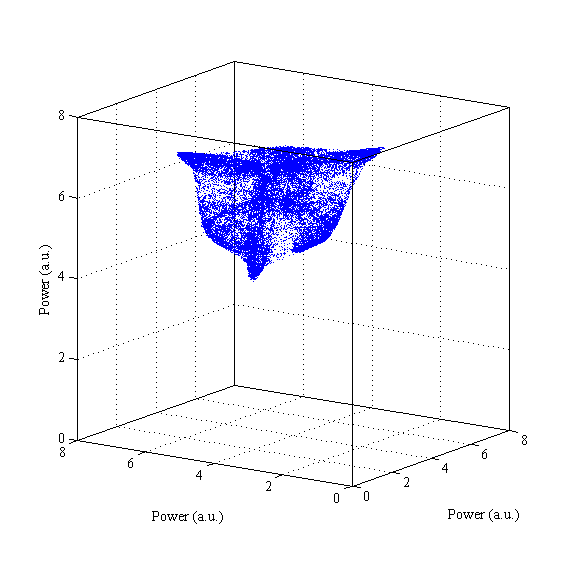

Supplement: Supplementary file 7 — Video S6 [file 41598_2017_1334_MOESM7_ESM.gif]

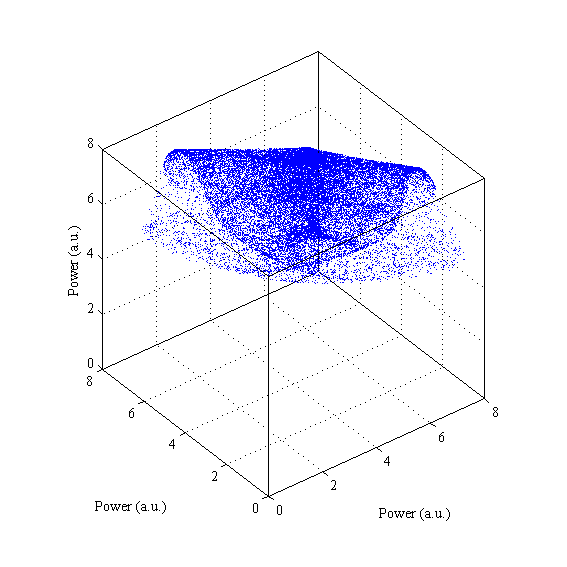

Supplement: Supplementary file 8 — Video S7 [file 41598_2017_1334_MOESM8_ESM.gif]

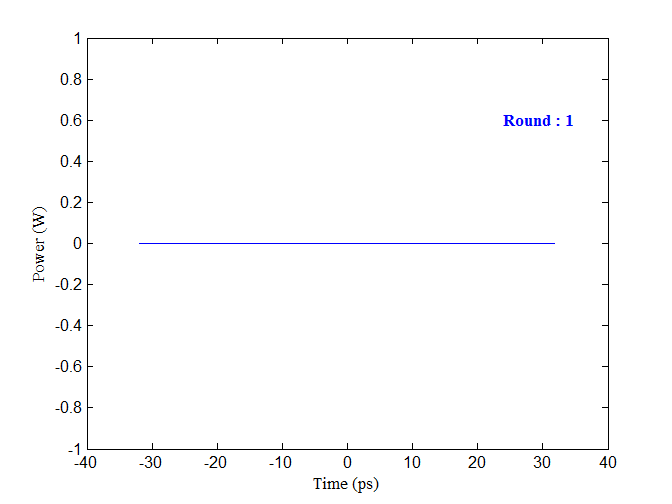

Supplement: Supplementary file 9 — Video S8 [file 41598_2017_1334_MOESM9_ESM.gif]

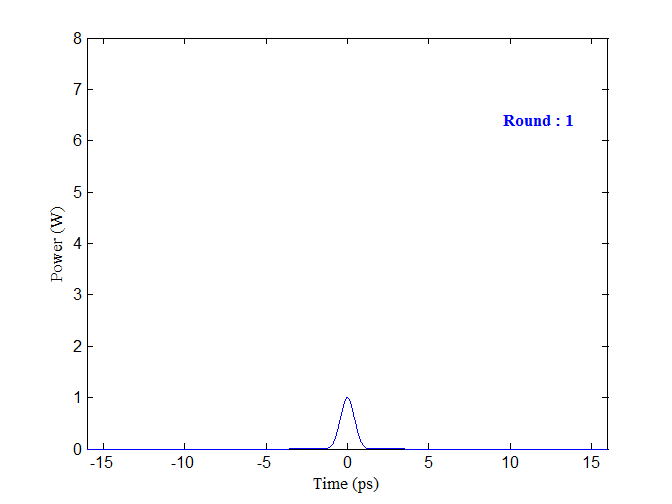

Supplement: Supplementary file 10 — Video S9 [file 41598_2017_1334_MOESM10_ESM.gif]

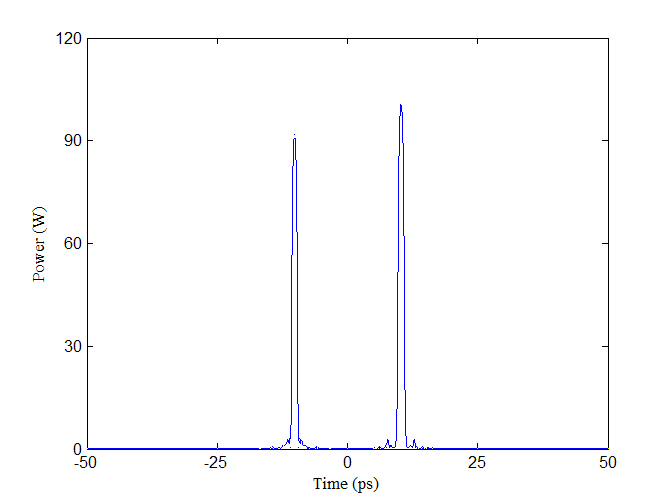

Supplement: Supplementary file 11 — Video S10 [file 41598_2017_1334_MOESM11_ESM.gif]
